# Effect of increasing cognitive activity participation on functional connectivities within default mode network– a randomized controlled trial

**DOI:** 10.1002/alz.088511

**Published:** 2025-01-09

**Authors:** Allen TC Lee

**Affiliations:** ^1^ The Chinese University of Hong Kong, Hong Kong China

## Abstract

**Background:**

We previously reported that increasing participation in Chinese calligraphy handwriting, one of the “Four Arts” traditionally mastered by intellectuals in China and currently widely practiced by older adults in the Chinese community, led to better working memory among individuals with subjective cognitive decline (but without mild or major neurocognitive disorder). Given that the default mode network (DMN) is closely correlated with the clinical symptoms and progression of Alzheimer’s disease, we aimed to investigate the effect of increasing calligraphy practice on the DMN.

**Method:**

Community‐living older adults who had been practicing Chinese calligraphy handwriting regularly (at least one hour of calligraphy practice per week) and experiencing subjective cognitive decline (but without mild or major neurocognitive disorder) were randomly allocated into either the control group, where they continued their usual practice of calligraphy, or the intervention group, where they doubled their amount of practice for 6 months. Functional connectivities within DMN were assessed before and after the intervention. This randomized controlled trial was funded by the Research Grants Council in Hong Kong (grant number: 24114519), registered with the Chinese Clinical Trial Registry (ChiCTR1900024433), and approved by the Clinical Research Ethics Committee of the Chinese University of Hong Kong (CREC Ref. No.: 2019.054).

**Results:**

By the end of 6 months, nearly all the functional connectivities that we examined within the DMN weakened in the control group, whereas those in the intervention group either strengthened or showed a lesser degree of weakening. Statistical significance was reached in the between‐group differences of the functional connectivities between medial prefrontal cortex and right lateral temporal cortex, medial prefrontal cortex and right inferior parietal lobe, left hippocampal formation and right lateral temporal cortex, and left hippocampal formation and right inferior parietal lobe.

**Conclusion:**

Increasing cognitive activity participation may have a positive neuromodulatory effect on functional brain network in older adults who are not yet having mild or major neurocognitive disorders. Our findings highlight the importance of staying cognitively active in late life for better brain health.

